# Movement-synchronized cerebellum rhythm coordinates multi-joint movements in young and elderly adults

**DOI:** 10.1242/bio.059776

**Published:** 2023-03-03

**Authors:** Keisuke Hirata, Hiroki Hanawa, Taku Miyazawa, Yohei Masugi

**Affiliations:** ^1^ Department of Rehabilitation, Faculty of Health Sciences, Tokyo Kasei University, Saitama, 350-1398, Japan; ^2^ Graduate Course of Health and Social Services, Graduate School of Saitama Prefectural University, Saitama, 343-8540, Japan; ^3^ Department of Rehabilitation, Faculty of Health Science, University of Human Arts and Sciences, Saitama, 339-8555, Japan; ^4^ Department of Physical Therapy, School of Health Sciences, Tokyo International University, Saitama, 350-1197, Japan

**Keywords:** Cerebellum, Coordination, Multi-joint movement, Cyclic, Rhythm

## Abstract

Rhythmic limb multi-joint movement like locomotion is controlled by intralimb coordination. Intralimb coordination changes entail immediate alterations in movement patterns and be related with cerebellum function. Synchronized cerebellum activity has known to modulate the frequency of walking, but not known the effect of only intralimb coordination. The purpose of this study was to reveal the effect of synchronized and unsynchronized cerebellum activity on the coordination of multi-joint movements of the unilateral leg in young and elderly people. To achieve our purpose, we applied synchronized and unsynchronized cerebellum transcranial alternating current stimulation during cyclic unilateral multi-joint movement by visual tracking task. The results showed that the reduction in comprehensive synchrony between targets and movements through trials had no significant differences under all stimulus conditions in young and elderly people. However, the reduction in variation of synchronization through trials was significantly smaller under the synchronized transcranial alternating current stimulation condition in both young and elderly groups. Variation of synchronization was remarkably reduced under the synchronized transcranial alternating current stimulation condition for the elderly group. This study showed that movement-synchronized cerebellum activity contributes to reducing fluctuations in movement synchrony by coordinating unilateral multi-joint movements. Moreover, this reduction was remarkable in the elderly group.

## Teaser

**Summary:** We investigated the effect of synchronized cerebellum activity on the coordination of multi-joint leg movements in young and elderly people. The synchronized activity was shown to reduce fluctuations in movement synchrony.

## INTRODUCTION

Rhythmic and discrete movements employ at least partially separate control mechanisms in the motor system (Howard, et al., 2011). A typical example of rhythmic movements of the limbs is joint movements of the lower limbs during walking. Coordination that controls multi-joint movements of the leg is involved in locomotion (Hinkel-Lipsker and Hahn, 2017). Multi-joint movement of one leg is controlled by intralimb (intersegmental) coordination (Aoi, et al., 2012; Reisman, et al., 2005). Intralimb coordination changes entail immediate alterations in movement patterns to accommodate the environmental dynamics on short timescales (Mawase, et al., 2013). This occurs due to changes in mechanical interactions with the environment and be affected by cerebellum function in patients with cerebellum lesions (Hoogkamer, et al., 2015). A previous study showed that patterned slow oscillatory brain stimulation matched with the frequency of the individual gait cycle entrained the rhythmic activities of the locomotor network including the cerebellum, leading to the modulation of natural human walking (Koganemaru, et al., 2020). As mentioned above, cyclic movement like locomotion is constructed by interlimb coordination (e.g. right and left legs), and intralimb coordination (e.g. hip and knee joint on unilateral leg) (Donaldson, et al., 2022). However, to our knowledge, no studies have directly demonstrated the effect of only intralimb coordination and synchronous activity of the cerebellum. In addition, motor coordination declines with age (Muehlbauer, et al., 2018). For example, movements become slower and less smooth when older adults move their shoulder and elbow joints simultaneously as opposed to performing single joint actions (Seidler, et al., 2010). The purpose of our study was to reveal the effect of synchronized and unsynchronized cerebellum activity on the coordination of multi-joint movements of the unilateral leg in young and elderly people. Experiments such as chasing tasks for a simple oscillated target [point-tracking (Summers, et al., 2018), finger-tapping (Odorfer, et al., 2019), and eye-tracking (Okada, et al., 2022)] have been used to demonstrate the relationship with cerebellum activity. To achieve our purpose using similar tasks, we applied synchronized and unsynchronized cerebellum activity during cyclic unilateral multi-joint movement to young and elderly adults.

## RESULTS

The Lilliefors test was performed on all the results of our study to check whether the data were normally distributed (*P*<0.01).

Table 1 shows the results of the Euclidean distance at the maximum and minimum target speeds by a paired *t*-test in the same condition within each group. The Euclidean distance at the maximum target speed had significantly higher values compared to the minimum target speed in all conditions within both young and elderly groups (*P*<0.05).

**Table 1.**
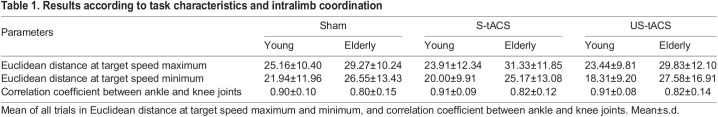
Results according to task characteristics and intralimb coordination

**Fig. 1.**
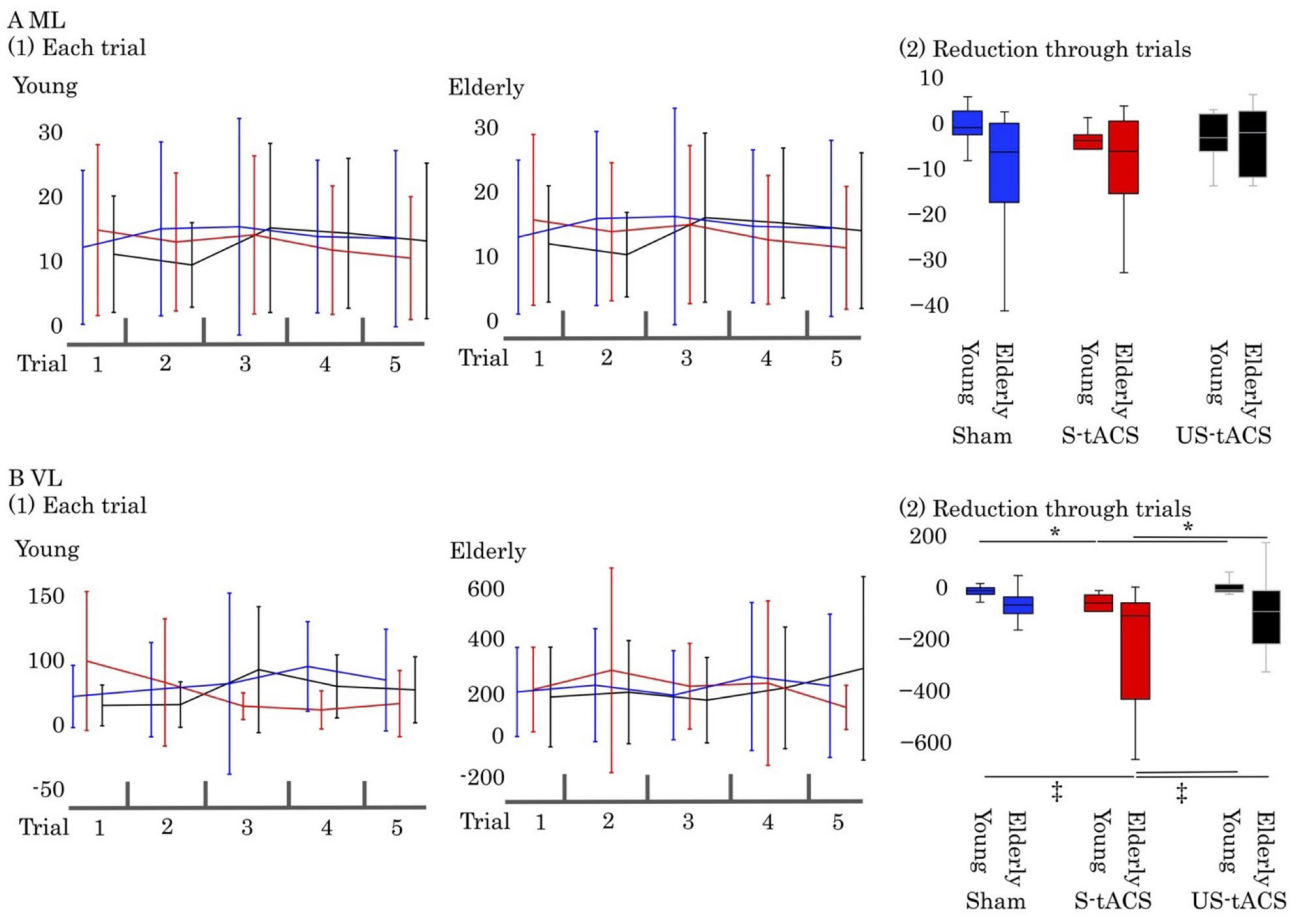
**Results of ML and VL.** (A1) The results of ML in each trial and (A2) the reduction of ML through trials. (B1) The results of VL in each trial and (B2) the reduction of VL through trials in sham (blue), S-tACS (red) and US-tACS (black). Error bar means the standard deviation, * means *P*<0.05 by one-way ANOVA within group and ‡ means *P*<0.05 by two-way ANOVA in all groups.

In both mean lag time (ML) and variation of lag time (VL), a two-way ANOVA revealed no significant interaction between the condition and group [*F* (2, 24)=0.43-93, *P*=0.39-65, η^2<^0.05]. The effect of group was significant [*F* (1, 2)=5.57-6.99, *P*<0.05, η^2=^0.07-0.66] while the effect of condition was not [*F* (2, 2)=0.31-2.89, *P*=0.06-73, η^2<^0.05]. Table 2 shows the results of ML and VL by a one-way ANOVA within each group. ML (Fig. 1A) had no significant difference between all conditions in both young (*P*=0.11-74) and elderly groups (*P*=0.97-9). VL (Fig. 1B) had a low value among all conditions in young (−74.39) and elderly (−323.05) groups. The reduction in comprehensive target-motor synchronism (ML, Fig. 1A) had no change in young and elderly groups (young: −3.39-1.05, elderly: −10.35-−9.36). On the other hand, the reduction in variation of synchronization through trials (VL, Fig. 1B) in S-tACS was changed clearly through the trials (young: −74.39, elderly: −323.05).

**Table 2.**
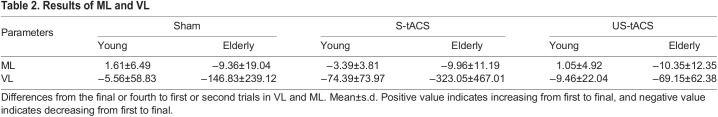
Results of ML and VL

Table 1 shows the results of the correlation coefficients between ankle and knee joints in each condition. A two-way ANOVA revealed no significant interaction between the condition and group [*F* (2, 24)=0.15, *P*=0.86, η^2<^0.01]. The main effect of group was significant [*F* (1, 2)=51.09, *P*<0.05, η^2=^0.11]. The main effect of condition was not significant [*F* (2, 2)=0.41, *P*=0.66, η^2<^0.01]. In all conditions, the elderly group (r=0.80-82) had significantly lower correlation coefficients than the young group (r=0.90-91). In the young group, VL had a significant difference between S-tACS and sham, US-tACS (*P*<0.05). In the elderly group, VL had a significant difference between S-tACS and US-tACS (*P*<0.05). There was an age-related difference in the variation of target-motor synchronism. In the elderly group, the standard deviation of VL was large, and a one-way ANOVA identified a significant difference only between S-tACS and US-tACS. However, there was a tendency for a larger reduction in VL through trials than in all conditions of the young group. A two-way ANOVA revealed that the elderly S-tACS group was significantly different to the young sham, young US-tACS, and elderly US-tACS groups (all *P*<0.05). Within the young group, the synchronized tACS condition showed significantly higher reduction through trials of synchrony variation through the trials than the other two conditions.

## DISCUSSION

This study aimed to investigate the effect of synchronized and unsynchronized cerebellum activity on the coordination of multi-joint movements of the unilateral leg in young and elderly people. The main outcome showed that the reduction in comprehensive synchrony between targets and movements through trials (ML) had no significant differences under all stimulus conditions in young and elderly people. However, the reduction in variation of synchronization through trials (VL) was significantly smaller under the synchronized transcranial alternating current stimulation condition (S-tACS) in both young and elderly groups. In particular, VL was remarkably reduced under the S-tACS condition for the elderly group.

### Task characteristics

The Euclidean distance at the maximum target speed had significantly higher values compared to the minimum target speed in all conditions within both young and elderly groups. An interesting feature of the results in all conditions and all groups was that Euclidean distance was decreased at the peak of the sine waveform of the target. Participants performed a common task in matching the timing of actual point with the turnaround point of the target oscillating. Matching the peak of the target's single oscillation was task-dependent. Therefore, the need to analyze the time difference between targets and movement peaks was revealed.

### Common effects of synchronized cerebellum activity on movement across ages

Both ML and VL had no significant interaction between the condition and group. The effect of group was significant while the effect of condition was not. ML had no significant difference between all conditions in both young and elderly groups. VL had a low value among all conditions in young and elderly groups. It is believed that tACS modulates cortical excitability by synchronizing or modulating the periodic rhythm of the brain (Antal and Herrmann, 2016; Vossen, et al., 2015). A previous study showed that tACS applied at around the frequency of the gait cycle can entrain the gait rhythm, suggesting a behavioral-relevant modulation of cerebellum-linked oscillators (Koganemaru, et al., 2020). We hypothesized that the reduction in comprehensive target-motor synchronism through trials (ML) would increase under the synchronous stimulus condition. However, the reduction in comprehensive target-motor synchronism had no change in young and elderly groups. On the other hand, the reduction in variation of synchronization through trials in S-tACS was changed clearly through the trials. The cerebellum contributes to motor error minimization processes (Popa, et al., 2012). As mentioned above, the cerebellum's contribution to intralimb coordination is obvious, which is performing a multi-joint coordination task of the unilateral leg (Hoogkamer, et al., 2015). tACS for motor cortex and cerebellum region improve dexterity during a visual following task (Miyaguchi, et al., 2013). In the task used this study, we interpret that the reduced variation of synchronization was due to the cerebellum predictively controlling the joint motion to the simple oscillating target.

Moreover, the task where movement is synchronized to the target using tACS (Vossen, et al., 2015) could be modulated from the temporal domain by adjusting the stimulation frequency to the individual average or peak frequency of the targeted oscillator (Wessel, et al., 2022). In the multi-joint movement task of this study, the cerebellum function could be involved in reducing the time error between the target and the actual movement. Intralimb coordination can be immediately changed in a shorter time span than interlimb coordination (Reisman, et al., 2005). During five trials of 30 s each, the change of comprehensive synchronization was not observed. However, intralimb coordination may have contributed to the reduction of synchrony variation in the short term of the five trials.

### Different effects of synchronized cerebellum activity with movement between ages

The correlation coefficients between ankle and knee joints in each condition had no significant interaction between the condition and group. The main effect of group was significant. The main effect of condition was not significant. In all conditions, the elderly group had significantly lower correlation coefficients than the young group. The intralimb coordination of multi-joints in unilateral leg could be affected by age, as stated in a previous study (Przybyla, et al., 2011). However, other studies have found that intralimb coordination is not age related (Aprigliano, et al., 2017). Because our task was a point-tracking task using multi-joint motion in simple leg movement and not in whole body movement, it is possible that the effect of age was more clearly apparent.

In the young group, VL had a significant difference between S-tACS and sham, US-tACS. In the elderly group, VL had a significant difference between S-tACS and US-tACS. There was an age-related difference in the variation of target-motor synchronism. Elderly group had large the standard deviation of VL and a significant difference only between S-tACS and US-tACS. However, there was a tendency for a larger reduction in VL through trials than in all conditions of the young group. The elderly S-tACS group was significantly different to the young sham, young US-tACS, and elderly US-tACS groups. Within the young group, the synchronized tACS condition showed significantly higher reduction through trials of synchrony variation through the trials than the other two conditions. It is natural to interpret this explanation as the effect of the synchronized tACS described above. In the elderly group, there was a particularly large reduction of synchrony variation in the synchronized tACS condition. The elderly group exhibited greater spatial and temporal movement variability, resulting in less consistent actions compared to young adults (Contreras-Vidal, et al., 1998; COOKE, et al., 1989). Such variability may arise from peripheral changes in the neuromuscular system (Faulkner, et al., 2007) or may arise from increased neural noise at the central nervous system level (Seidler, et al., 2010; Welford, 1958). This suggests that the impact of synchronized tACS may be great for the elderly who have a larger synchrony variation. Cerebellum activity that intermittently deviates from movement may mislead the motor synchrony for the elderly. The unsynchronized tACS condition in the elderly group was not significantly different from all conditions of the young group and the synchronized tACS was not significantly different from the sham condition within the elderly group.

### Implication

The sham condition led to similar reduction of synchrony variation through trials as the synchronized tACS condition in the elderly group. This reduction of synchrony variation in the elderly group can be interpreted as a simple effect of the task. Environmental factors such as exercise, training, injuries, and rehabilitation change neural plasticity by varying degrees in different cortical regions. In humans, intentional exercise and cortical plasticity are closely related; exercise contributes to better memory by elevating brain-derived neurotrophic factor (Vaynman, et al., 2006). In terms of age effects, motor learning skill differences between young and elderly people reflect differences in the ability of the central nervous system to reduce neural noise and coordinate agonist-antagonist muscle activities (Christou, et al., 2007). From the training, the elderly group demonstrated ‘motor plasticity’, the ability to acquire new motor skills, to an extent similar to that of young adults (Voelcker-Rehage and Willimczik, 2006). From the multi-joint movement task, it is interpreted that the short-term changes in intralimb coordination led to the training effect. The present study demonstrated the clinical implication of the synergistic effect of multi-joint movement training and promoting synchronized cerebellum activity. The elderly have poor motor coordination of the leg when controlling the swing foot (Yamagata, et al., 2019a) and are likely to fall in the future (Yamagata, et al., 2019b). For example, coordinated flexion of the knee and ankle joints on the unilateral leg could reduce fall risk by ensuring clearance in the swing phase during walking. Neuromodulation to promote movement-synchronized cerebellum activity may enhance the training effect of a unilateral leg multi-joint exercise in the elderly.

A limitation of this study was that the results for task difficulty, which produces a large difference in comprehensive synchrony between the elderly and the young, are unclear. Even if a task has high difficulty, it is debatable whether there is no effect on comprehensive synchrony. In the future, verification through tasks with a higher difficulty are required. However, since the multi-joint movement task is expected to be difficult for the elderly and patients, we believe that it is always necessary to set the level of the task according to the ability of the participant.

### Conclusion

This study showed that movement-synchronized cerebellum activity contributes to reducing fluctuations in movement synchrony by coordinating unilateral multi-joint movements. Moreover, this reduction was remarkable in the elderly group. We present a clinical application of modulating cerebellum activity during interventions to improve motor coordination in the elderly and sick. In addition, it was shown that focusing on changes in variation of temporal synchrony of cyclic movements may serve as an indicator of decreased motor coordination.

## MATERIALS AND METHODS

### Participants

Twelve healthy young adults (nine females; age: 23.8±4.1 years) and 17 healthy elderly adults (nine females; age: 72.8±5.5 years) with no physical problems and no neuromuscular disease were included. The sample size was comparable to those of previous studies of similar experiments (Koganemaru, et al., 2020; Miura, et al., 2018). We planned to exclude participants with a poor understanding of the experimental task, but no participant was excluded. All experiments were conducted after obtaining informed consent from the participants in accordance with the Declaration of Helsinki and approval from the affiliated institutional review board.

### Recording procedure and software setting

**Fig. 2.**
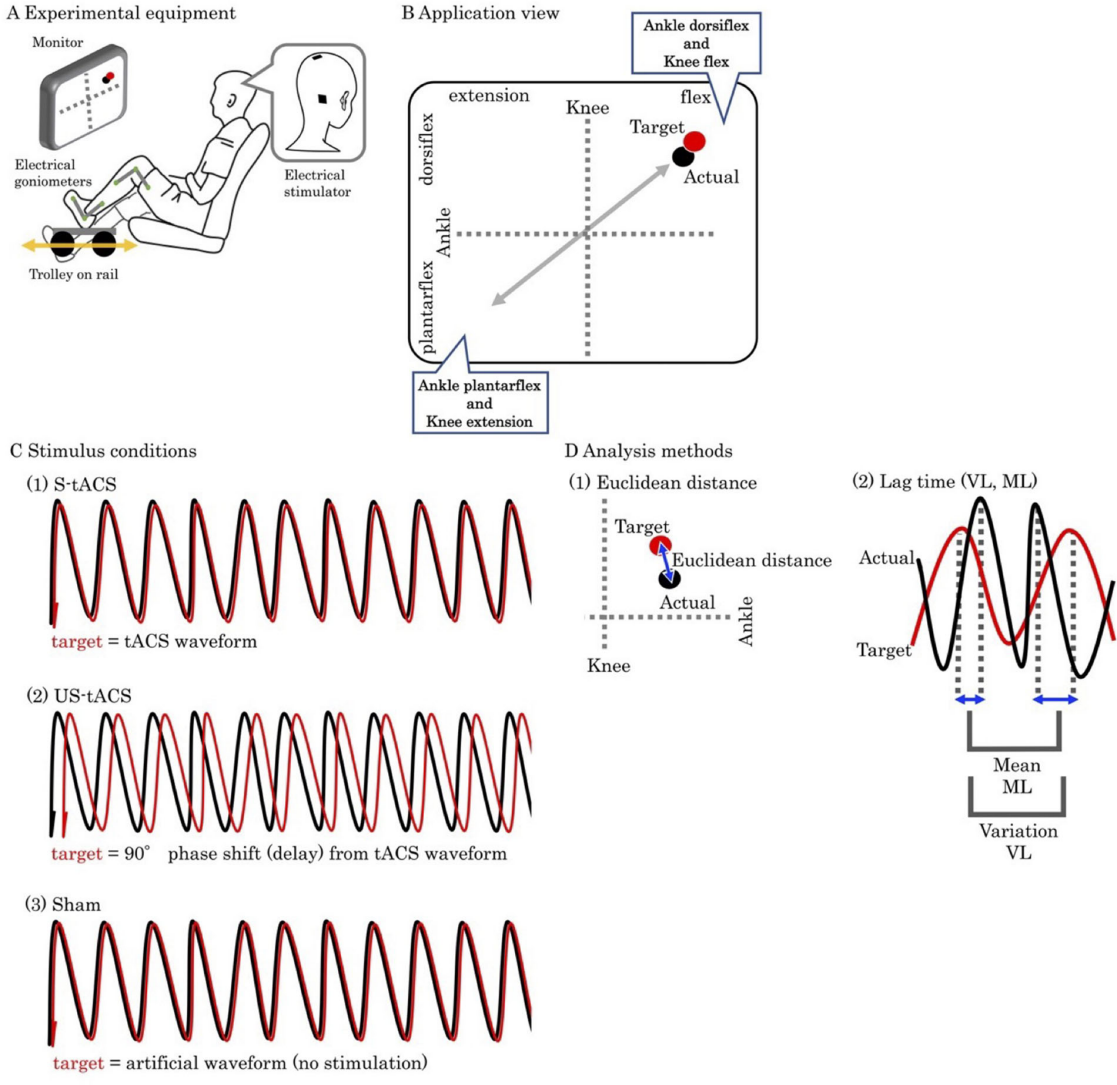
**Experiment and analysis methods.** (A) Experimental equipment. Participant sat looking at the monitor with the electrical stimulator to collabulary and put the foot on the trolley. (B) Monitor view of a two-dimensional coordinate. x axis was the knee joint with the flexion (extension) as positive (negative) and y axis was the ankle joint with the dorsi flexion (plantar flexion) as positive (negative). x and y axis were normalized by each participant's range of motion during the task. Participants tracked the ‘target’ point (red) with the ‘actual’ point (black) by multi-joint movement (knee and ankle). (C) Three stimulus conditions were: (1) synchronized tACS with the target, (2) unsynchronized tACS with the target (90° phase delay from the target), and (3) sham stimulation. (D) Analysis methods of (1) Euclidean distance between actual and target points, and (2) lag time of mean (ML) and variation (VL) between actual and target points.

Participants sat in the chair with the experimental side leg on the trolley. The experimental side leg was the dominant side when kicking a ball (Chapman and Chapman, 1987). To avoid misalignment, the trolley slid on a rail and the experimental foot was strapped to the trolley with a band (Fig. 2A).

Ankle and knee joint flex/extension angles were assessed by electrical goniometers for each joint (AC-GM150, 4Assist Co., Ltd, Japan). The current voltage from the goniometers and waveform from the stimulator were input into the computer through the A/D convertor (NI USB-6343BNC, National Instrument Corp., USA) by sampling at 5000 Hz.

The software was constructed by LabVIEW (2021, National Instrument Corp., USA) as follows. A two-dimensional coordinate centered at (50, 50) from 0 to 100 was displayed on the monitor (Fig. 2B). The knee joint is shown on the x axis with the flexion (extension) assigned positive (negative) values. The ankle joint is shown on the y axis with the dorsi flexion (plantar flexion) assigned positive (negative) values. The x and y axis were normalized by each participant's range of motion during the task. Therefore, on the coordinates of the monitor, the condition of the participant's foot and knee joints was displayed as the single ‘actual’ point (black). If participants successfully performed the task described below, ‘task’, the actual point moved back and forth from (0, 0) to (100, 100). Moreover, the ‘target’ point (red) was oscillated from (0, 0) to (100, 100) at 0.4 Hz sine waveform according to waveform from stimulator or artificial waveform (sham stimulation) by LabVIEW.

### Task, stimulus conditions, and cerebellum stimulation

Participants were asked to track the target point and move the actual point for 5 min while undergoing the tACS conditions described (‘cerebellum stimulation’ below). To move the actual point, the participant would repeat the combination of knee flexion and ankle dorsi flexion, and the combination of knee extension and ankle plantar flexion. Five trials of a 30 s tracking task with 15 s rest were performed for all three stimulus conditions.

Three stimulus conditions (Fig. 2C) were performed in a random order at least 30 min apart on a single day: (1) synchronized tACS with the target (S-tACS), (2) unsynchronized tACS with the target (US-tACS) and (3) sham stimulation. S-tACS matched the target with the tACS waveform. US-tACS matched the target with a 90-degree phase shift (delay) from the tACS waveform. Sham had no tACS that matched the target with artificial waveform. We did not tell the participants the order of the conditions and, after completing all conditions, we confirmed with them that there were no differences in sensation between the conditions during the experiment.

The electrode (5×5 cm) was centered 3 cm experimental side-lateral from the inion, and the electrode (3×3 cm) was placed 2 cm opposite side-lateral from the vertex (Koganemaru, 2020) using a NeuroConn DC Stimulator (Ilmenau, Germany, Fig. 2A). The tACS was a peak-to-peak stimulation current of 2 mA (from −1 mA to +1 mA) with 60 s fade-in and fade-out periods. Recorded tACS currents were filtered offline using a zero-phase bandpass at the cut-off frequencies of 0.4 and 2 Hz.

### Analysis

All recorded waveforms were downsampled from 5000 Hz to 100 Hz and the first 5 s were removed from the 30 s data. Joints angles were smoothed by a forward and backward second-order Butterworth low-pass filter with a cut-off frequency of 5 Hz.

First, we assessed the timing of tracking error to verify that participants performed the common task of matching the actual point to the turnaround point of the target oscillating. Euclidean distance between the actual and target points was compared at target speed maximum and minimum in each condition within each group (Fig. 2D1).

Second, we assessed the correlation coefficients between ankle and knee joints as the parameter of the intralimb coordination. The means of correlation coefficients were calculated by each cycle in each condition.

Third, we assessed the parameters of the temporal synchronization as follows (Fig. 2D2). We calculated the lag (delay or lead) time between the peak timings of the target and actual points. Multiple biological coordination studies reveal that the coordination task includes reactive (responding to a stimulus pattern) and anticipatory (responding with a stimulus pattern) effects (Engström, et al., 1996). We assessed the difference of mean lag time from the final or fourth to first or second trials (ML) to quantify the comprehensive lag reduction through trials. Similarly, we assessed the difference of variation of the lag time from the final or fourth to first or second trials (VL) to quantify the extent of lag wandering reduction through trials (Miura, et al., 2018). Mean and variation were calculated in each phase, and we removed statistical outliers using mean +/-2 SD. All parameters were calculated using the difference between the larger one of the first and second trials and the smaller one of the fourth and last trials as the amount of change through trials in each condition.

### Statistics

Two-way repeated measures ANOVA was used to identify statistically significant interactions in all parameters between conditions (sham, S-tACS, and US-tACS) and groups (young and elderly). Within-subjects one-way repeated measures ANOVA was used to identify statistically significant differences among the different conditions. If analysis of variance revealed significant main effects or interactions, Tukey's post-hoc comparisons were carried out to identify significant differences among variables. All analyses were performed with a significance level set at *P*=0.05 using MATLAB (2022a, MathWorks Inc., MA, USA). We have also provided eta-squared (η2) effect sizes to supplement the interpretation of the results.

## Supplementary Material

10.1242/biolopen.059776_sup1 Supplementary information
