# Supplementary material for: Movement-synchronized cerebellum rhythm coordinates multi-joint movements in young and elderly adults
Source: Biol Open. 2023 Mar 3;12(3):bio059776. doi: 10.1242/bio.059776 (PMC10003071; doi:10.1242/bio.059776)
Supplement: Supplementary information [file biolopen-12-059776-s1.pdf]

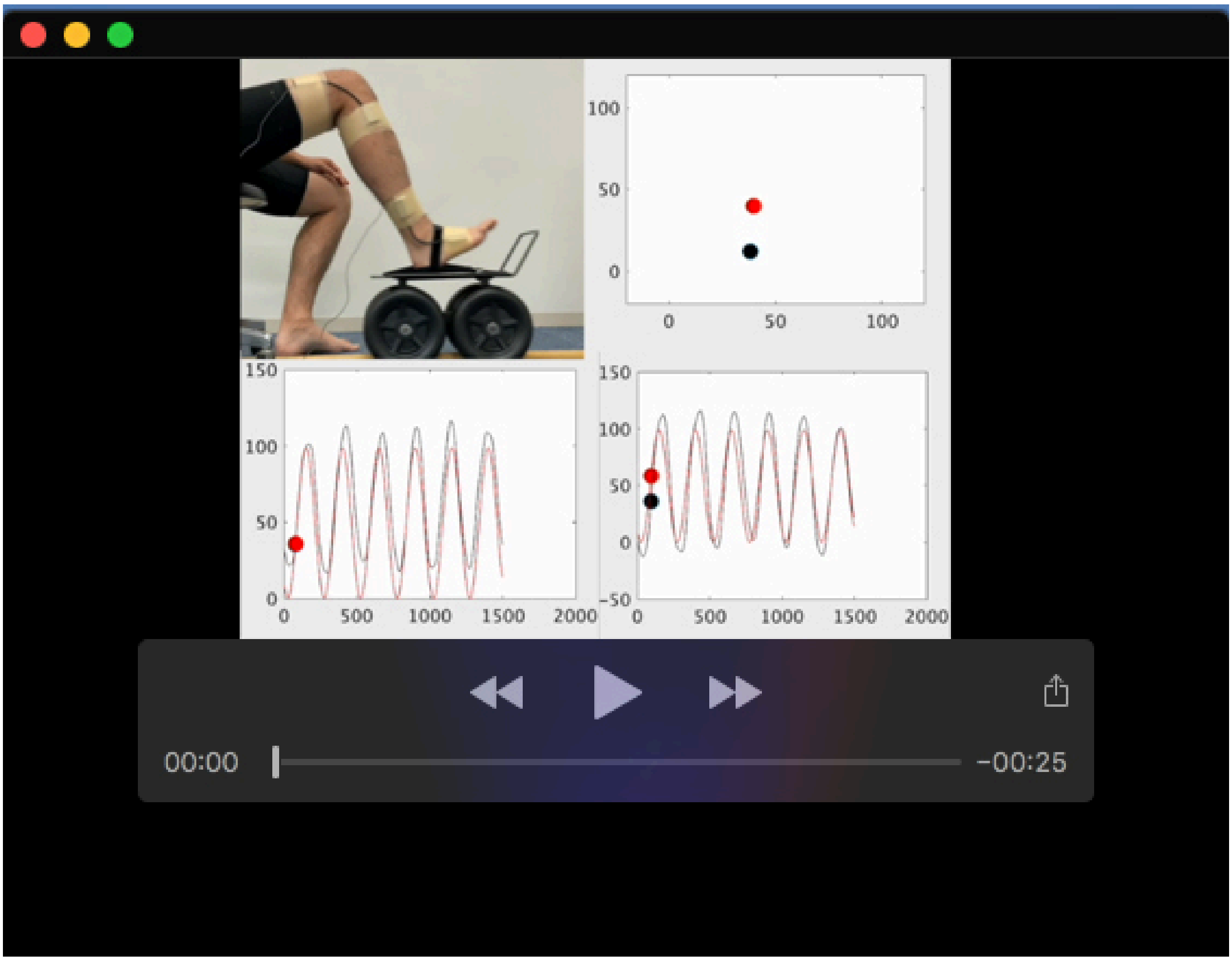

Movie 1. Right above: A two-dimensional coordinate centered at (50, 50) from 0 to 100 was displayed on the monitor (right above). The knee joint is shown on the x axis with the flexion (extension) assigned positive (negative) values. The ankle joint is shown on the y axis with the dorsi flexion (plantar flexion) assigned positive (negative) values. Therefore, on the coordinates of the monitor, the condition of the participant's foot and knee joints was displayed as the single "actual" point (black). If participants successfully performed the task, the actual point moved back and forth from (0, 0) to (100, 100). Moreover, the "target" point (red) was oscillated from (0, 0) to (100, 100) at 0.4 Hz sine waveform according to waveform from stimulator or artificial waveform (sham stimulation). Left above: To move the actual point, the participant would repeat the combination of knee flexion and ankle dorsi flexion, and the combination of knee extension and ankle plantar flexion. Right bottom: Knee joint motion and target point. Left bottom: Ankle joint motion and target point.

Biology Open • Supplementary information
